# Proline-Based Cyclic Dipeptides from Korean Fermented Vegetable Kimchi and from *Leuconostoc mesenteroides* LBP-K06 Have Activities against Multidrug-Resistant Bacteria

**DOI:** 10.3389/fmicb.2017.00761

**Published:** 2017-05-02

**Authors:** Rui Liu, Andrew H. Kim, Min-Kyu Kwak, Sa-Ouk Kang

**Affiliations:** Laboratory of Biophysics, School of Biological Sciences, and Institute of Microbiology, Seoul National UniversitySeoul, South Korea

**Keywords:** cyclic dipeptides, kimchi, Korean fermented foods, lactic acid bacteria, *Leuconostoc mesenteroides* LBP-K06

## Abstract

*Lactobacillus plantarum* and *Leuconostoc mesenteroides* play a prominent role as functional starters and predominant isolates in the production of various types of antimicrobial compound-containing fermented foods, especially including kimchi. In the case of the bioactive cyclic dipeptides, their racemic diastereomers inhibitory to bacteria and fungi have been suggested to come solely from *Lactobacillus* spp. of these strains. We previously demonstrated the antifungal and antiviral activities of proline-based cyclic dipeptides, which were fractionated from culture filtrates of *Lb. plantarum* LBP-K10 originated from kimchi. However, cyclic dipeptides have not been identified in the filtrates, either from cultures or fermented subject matter, driven by *Ln. mesenteroides*, which have been widely used as starter cultures for kimchi fermentation. Most importantly, the experimental verification of cyclic dipeptide-content changes during kimchi fermentation have also not been elucidated. Herein, the antibacterial fractions, including cyclo(Leu-Pro) and cyclo(Phe-Pro), from *Ln. mesenteroides* LBP-K06 culture filtrates, which exhibited a typical chromatographic retention behavior (t_R_), were identified by using semi-preparative high-performance liquid chromatography and gas chromatography-mass spectrometry. Based on this finding, the proline-based cyclic dipeptides, including cyclo(Ser-Pro), cyclo(Tyr-Pro), and cyclo(Leu-Pro), were additionally identified in the filtrates only when fermenting Chinese cabbage produced with *Ln. mesenteroides* LBP-K06 starter cultures. The detection and isolation of cyclic dipeptides solely in controlled fermented cabbage were conducted under the control of fermentation-process parameters concomitantly with strong CDP selectivity by using a two-consecutive-purification strategy. Interestingly, cyclic dipeptides in the filtrates, when using this strain as a starter, increased with fermentation time. However, no cyclic dipeptides were observed in the filtrates of other fermented products, including other types of kimchi and fermented materials of plant and animal origin. This is the first report to conclusively demonstrate evidence for the existence of antimicrobial cyclic dipeptides produced by *Ln. mesenteroides* in kimchi. Through filtrates from lactic acid bacterial cultures and from fermented foods, we have also proved a method of combining chromatographic fractionation and mass spectrometry-based analysis for screening cyclic dipeptide profiling, which may allow evaluation of the fermented dairy foods from a new perspective.

## Introduction

The beneficial effects of lactic acid bacteria (LAB) have been steadily demonstrated, such as competitive exclusion of enteric pathogens, tumor suppression through cell-mediated immunity, and especially host defense enhancement by antimicrobial productions (Naidu et al., 1999). Among these pivotal behaviors, antimicrobial activities have closely associated with secretory metabolic products found in LAB themselves and in their culture filtrates (Naidu et al., 1999; Ross et al., 2002; Rouse and Van Sinderen, 2008). They are proficient regulators, governing the influx of the environmental and pathogenic or spoilage-causing microbes in fermented foods: Gram-positive and -negative bacteria—e.g., *Arthrobacter* sp., *Acinetobacter* sp., *Bacillus subtilis, Escherichia coli, Listeria monocytogenes, Pseudomonas aeruginosa, Staphylococcus aureus—*(Sarika et al., 2012), and fungi—e.g., *Aspergillus nidulans, Penicillium commune, Fusarium sporotrichioides, Rhodotorula mucilaginosa—*(Magnusson et al., 2003; Digaitiene et al., 2012). Additionally, *Lactobcillus casei* DN-114 001 culture supernatants and *Lb. paracasei* ST284BZ bacteriocins bacST284BZ have antiviral activities against infections by changing glycosylation or galactosylation of rotavirus receptors in HT-29 cells (Freitas et al., 2003a,b) and herpes simplex virus Type 1, despite its elusive mechanism of actions (Todorov et al., 2008). Thus, the significance of LAB-produced secondary metabolites has been focused, so far, mainly on low-molecular-weight molecules seemingly representative of bacteriocins or bacteriocin-like substances and peptidyl or non-peptidyl small molecules (Messens and De, 2002). For this reason, amino acid (AA) metabolism renders bulk contribution to physiological events sufficiently amicable to permit small peptide and protein biosynthesis, pH modulation, metabolic energy/redox balance alteration, and stress resistance by various types of intermediate metabolites, and most importantly, whether differently in raw material fermentation or in culture media fermentation (Fernádez and Zúñiga, 2006; Rouse and Van Sinderen, 2008). For example, when using complex media, *Lb. plantarum* displays absolute requirements for typical types of essential proteogenic (i.e., isoleucine, leucine, valine, lysine, tryptophan, and threonine) and non-essential proteogenic (i.e., glutamic acid and cysteine) AAs (Saguir and Manca De Nadra, 2007). However, the combination of essential AAs with a simple composition allows for *Ln. mesenteroides* growth regulated by several types of branched-chain AA transport systems (Foucaud et al., 1997, 2001).

Non-bacteriocin molecules, commonly including organic acids, AAs, fatty acids, diacetyl, and hydrogen peroxide, have been generally characterized as antimicrobial effectors against bacteria, fungi, and viruses (Vandenbergh, 1993; Naidu et al., 1999; Rouse and Van Sinderen, 2008). Particularly, the antimicrobial small compounds from *Lactobacillus* spp. associated with fermented products have been steadily emphasized. The reutericyclin of *Lb. reuteri* LTH2584 has been shown to have antibacterial activity against Gram-positive bacteria (Gänzle et al., 2000). In culture filtrates from *Lactobacillus plantarum* VTT E-78076, benzoic acid, methylhydantoin, mevalonolactone, and cyclo(glycyl-l-leucyl) have been found to be active against bacteria and fungi (Niku-Paavola et al., 1999). Furthermore, significant antifungal properties have been demonstrated in *Lb. casei* AST18 producing compounds—e.g., cyclo(Leu-Pro), 2,6-diphenyl-piperidine, and 5,10-diethoxy-2,3,7,8-tetrahydro-1H,6H-dipyrrolo[1,2-a;1′,2′-d]pyrazine—(Li et al., 2012), *Lb. plantarum* MiLAB 393 generating substrates—e.g., cyclo(l-Phe-l-Pro), cyclo(l-Phe-trans-4-OH-l-Pro), and 3-phenyllactic acid—(Ström et al., 2002), and *Lb. plantarum* IMAU10014 secreting metabolites—e.g., benzoic acid and benzeneacetic acid—(Wang et al., 2012).

Considering these small substances, several types of 2,5-diketopiperazines, cyclic dipeptides (CDPs), and their stereoisomers have been suggested to exhibit potent antimicrobial activities (Witiak and Wei, 1990; Prasad, 1995; Dinsmore and Beshore, 2002; Huang et al., 2010, 2014). Bioactive CDPs function due to their structural chirality and varied side chains; thus, they serve as attractive scaffolds for drug design (Borthwick, 2012). These dipeptidyl cyclic ring closures have been suggested for decades as signal molecules, reducing virulence-factor production and strongly inhibiting microbial growth (Campbell et al., 2009; Huang et al., 2010; Sauguet et al., 2011). For example, cyclo(ΔAla-l-Val), cyclo(l-Pro-l-Tyr), and cyclo(l-Phe-l-Pro) activate a LuxR-based *N*-acyl homoserine lactones biosensor, one of the most intensively investigated families of intercellular signal molecules (Holden et al., 1999). Cyclo(His-Pro), which have also shown potential therapeutic utility in an array of neurological and peripheral inflammatory diseases, are also known as a group of hormone-like molecules appearing in organisms from bacteria to humans (Bellezza et al., 2014).

Our recent reports have demonstrated the bioactivity of proline-based CDPs, including *cis*-cyclo(l-Leu-l-Pro), *cis*-cyclo(l-Phe-l-Pro), and *cis*-cyclo(l-Val-l-Pro). These compounds, fractionated from culture filtrates of *Lb. plantarum* LBP-K10, were inhibitory to the proliferation of influenza A virus (H3N2) (Kwak et al., 2013) and plant and human pathogenic fungi (Kwak et al., 2014a). *Lb. plantarum* LBP-K10 was observed to be a key antimicrobial isolate from kimchi, together with other potent *Leuconostoc* spp., *Lactobacillus* spp., *Weissella* spp., and a *Lactococcus lactis* (Kwak et al., 2013). All these strains have already been recognized to be relevant as the predominant LAB during kimchi fermentation (Lee et al., 1999; Yang and Chang, 2010; Kwak et al., 2014a,b). Particularly, *Ln. mesenteroides* is an important starter culture strain capable of performing uniform high quality commercial kimchi, which has been generally made from Chinese cabbage (*Brassica rapa* subsp. *pekinensis*) (Lee et al., 1992; Jung et al., 2014).

However, despite the importance of exactly which bioactive metabolites appear in starter culture strains and vegetables for kimchi fermentation, no available studies examined CDPs particularly, or their derivatives with potent and selective antimicrobial activity associated with the specific bacterial fermentation products. Most prominently, the experimental evidence for CDP production by *Ln. mesenteroides* is completely elusive in culture filtrates and fermented materials, as with kimchi. Therefore, this study considers the novel possibility that antimicrobial CDPs in the filtrates from *Leuconostoc* cultures and from fermented kimchi produced with or without *Ln. mesenteroides* LBP-K06 starter cultures. Interestingly, we failed to find CDPs in the whole filtrates from non-starter kimchi and other fermented foods of plant or animal origin. We only detected CDPs in Chinese cabbage kimchi (CCK) produced with *Ln. mesenteroides* starter cultures. Consequently, we identified two CDPs—cyclo(Leu-Pro) and cyclo(Phe-Pro)—in the culture filtrates from *Ln. mesenteroides* LBP-K06 and three proline-based CDPs—cyclo(Ser-Pro), cyclo(Tyr-Pro), and cyclo(Leu-Pro)—in starter kimchi using high-performance liquid chromatography (HPLC) followed by gas chromatography–mass spectrometry (GC-MS). For the first time, this study demonstrates isolated antimicrobial CDPs and their relative amounts in filtrates from bacterial cultures and from kimchi fermented with *Ln. mesenteroides*.

## Materials and methods

### Strains and culture conditions

All bacterial strains used in this study are listed in Table 1. *Ln. mesenteroides* LBP-K06 and *Lb. plantarum* LBP-K10 were routinely cultured on modified de Man, Rogosa, and Sharpe (MRS without beef extract) agar (De Man et al., 1960) in a broth at 30°C for 3 days. The culture was stored anaerobically on 1.0% MRS agar plates at 5°C. *Ln. mesenteroides* LBP-K06 was used for fermenting Chinese cabbage, as described previously, with some modifications (Cheigh and Park, 1994).

**Table 1:** **LAB strains isolated from three types of Korean traditional fermented vegetables and Gram-positive, Gram-negative, and multidrug-resistant bacteria used in this study**.

**Strain**	**Types or strains**	**Source or reference**
**LAB STRAINS**		
*Ln. mesenteroides* LBP-K06	Original isolate from fermented Chinese cabbage	This study
*Lb. plantarum* LBP-K10	Original isolate from fermented Chinese cabbage	Kwak et al., 2013, 2014a
**BACTERIAL INDICATOR STRAINS**		
Gram-positive bacteria	*Bacillus subtilis*	This Study
	*Staphylococcus aureus*	This Study
Gram-negative bacteria	*Escherichia coli*	This Study
	*Salmonella Typhimurium*	This Study
**MULTIDRUG-RESISTANT BACTERIA**		
**Gram-positive bacteria**		
*S. aureus* 11471	oxacillin-resistant *S. aureus* (ORSA) 11471, which is resistant to beta-lactam antibiotics, including penicillins (methicillin, dicloxacillin, nafcillin, and oxacillin) and cephalosporins	This study, KNIH^a^
**Gram-negative bacteria**		
*S. Typhimurium* 12219	*S. Typhimurium* 12219, which is resistant to ACSSuT (ampicillin, chloramphenicol, streptomycin, sulphonamides, and tetracycline)	This study, KNIH^a^

a *This was supported by the Korea National Institute of Health*.

To observe the antibacterial activity of the isolated substances and filtrate fractions from the bacterial cultures and the fermented kimchi, respectively, we used Gram-positive and -negative bacterial indicators and multidrug-resistant bacteria in this study (Table 1). All bacterial pathogens were supplied by the Korea National Institute of Health.

### Preparation of fermented foods and their filtrates

The filtrates from Chinese cabbage were obtained by controlled and spontaneous fermentation with and without inoculation by *Ln. mesenteroides* LBP-K06 as a starter strain, respectively, as proposed previously (Cheigh and Park, 1994; Jung et al., 2011, 2012, 2014), with some modifications. The spontaneously fermented Chinese cabbage was obtained at the late kimchi fermentation stage. In the case of the controlled fermentation for the production of kimchi made from Chinese cabbage, after it was fermented until the initial stage, an additional fermentation was performed at 25°C for 72 h until the middle kimchi fermentation stage. Other types of kimchi, including young radish (YRK), water-based radish (WRK), and sliced radish (SRK), were spontaneously fermented without any inoculum to the late kimchi fermentation phase described previously (Cheigh and Park, 1994). Kimchi filtrates were prepared from all types of fermented products by freeze drying, grounding to a powder, and filtering through a #80-mesh (180 micron) sieve. The resulting powder of fermentation filtrates, which was dissolved in sterilized distilled water and filtered with a 0.22 μm-cellulose acetate membrane, was subjected to methylene chloride (HClO_4_) extraction for further HPLC fractionation.

All other fermented products were prepared as follows. The Korean traditional fermented foods of plant origin, including cheonggukjang (fast-fermented bean paste), doenjang (soybean paste), *B. subtilis* nattō, and soy sauce were prepared according to the proposed methods (Shin and Jeong, 2015). Additionally, pickled or salted shrimp and clams, fermented products of animal origin, were purchased from the traditional market, Togulsaeujeot-gil (underground tunnel in Gwangcheon province), in Chungcheongnam-do in Korea. The fermentation filtrates from the above fermented products were obtained by centrifugation, lyophilization, HClO_4_ extraction, and filtration, respectively, as described earlier.

### HPLC fractionation

The filtrate fractionation was performed by HPLC as proposed previously (Kwak et al., 2013). The resulting primary fractions were consecutively lyophilized, extracted with HClO_4_, and further fractionated by changing the mobile-phase compositions so as to give an improved quantitative chromatographic fractionation. The filtered samples were separated using a semi-preparative HPLC system (Agilent, USA) with a semi-preparative Hypersil octadecyl silica (ODS) C18 reverse-phase column (9.4 × 250 mm, Agilent, USA) and ChemStation HPLC software (Kwak et al., 2013). The initial mobile phase was 67% water, 3% acetonitrile, and 30% methanol for 45 min, and the wavelengths for observing the corresponding chromatograms were 210, 260, and 280 nm, respectively. Each fraction was collected and concentrated by lyophilization to obtain a powder for antibacterial activity determination and GC-MS analysis.

### Antibacterial assays

The antibacterial activity against Gram-positive indicators, Gram-negative bacterial indicators, and multidrug-resistant bacteria was examined every 24 h after seed inoculation. The dilution method was used to determine the minimum inhibitory concentration (MIC) of antimicrobial substances (Huys et al., 2002; Paulo et al., 2010). To evaluate antimicrobial activity, we prepared the identified CDPs and filtrate fractions from LAB cultures and from various types of fermented products and tested them against certain pathogenic bacteria.

### Mass analysis

To perform electron ionization (EI) and chemical ionization (CI) of each fraction, we used GC-MS (Agilent, Germany), as described previously (Kwak et al., 2013). A chromatographic system consisting of an Agilent 6890 series GC equipped with a 7679 series automatic liquid sampler was used. Mass analysis was conducted using a high-resolution mass spectrometer (JEOL JMS-700, Japan).

### Statistical analysis

Results are presented as means ± standard deviation (*SD*). The statistical significance of the differences was tested using Student's *t*-test in Microsoft Office Excel (2013). For all comparisons, values of *p* < 0.05 (^*^) were considered statistically significant.

## Results

### Identification of proline-containing CDPs found in the culture filtrate from *Ln. mesenteroides* LBP-K06 isolated from kimchi

**Figure 1:**
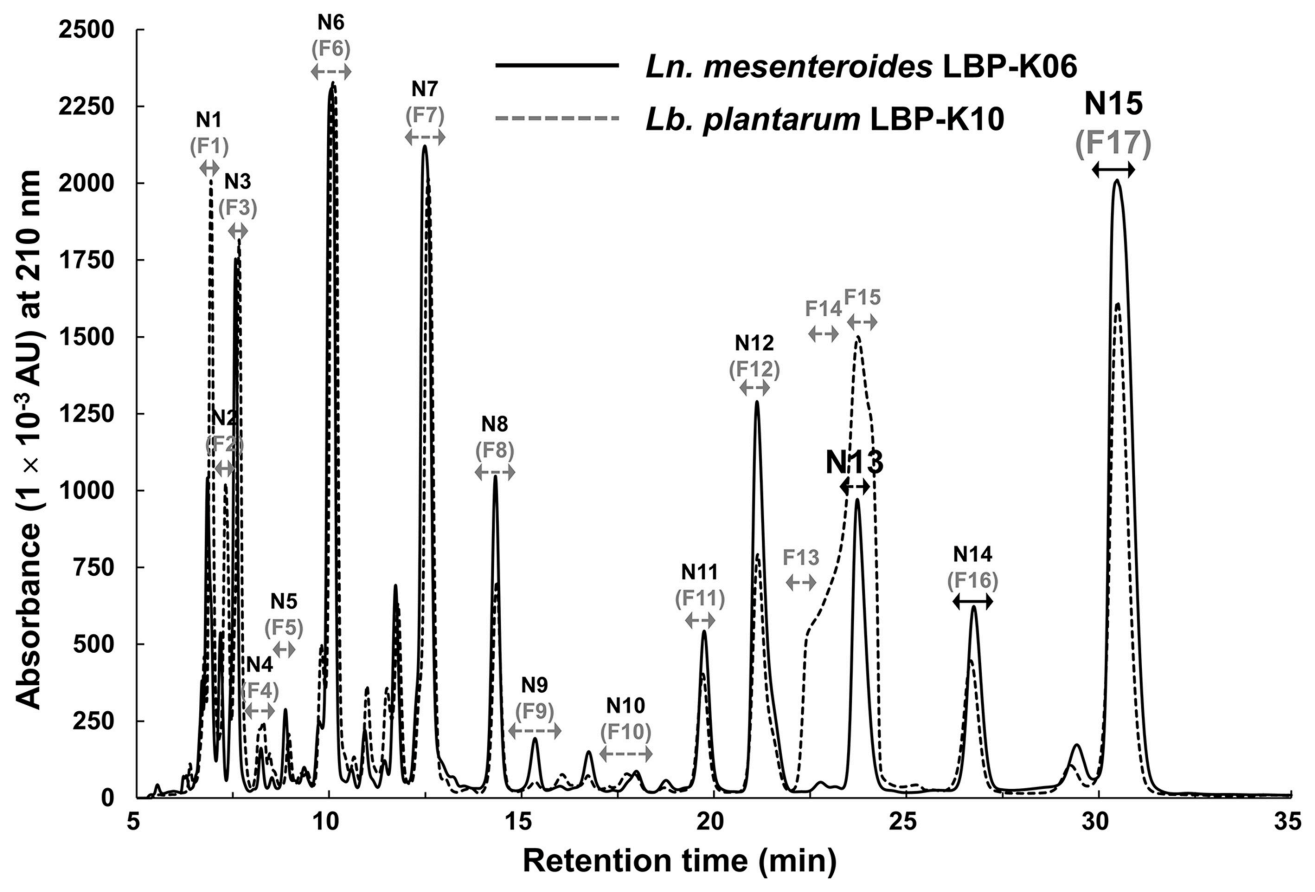
**The chromatographic separation of N13 and N15 by *Ln. mesenteroides* LBP-K06 compared with corresponding fractions of *Lb. plantarum* LBP-K10. HPLC profiles of both strains were performed at a wavelength of 210 nm**. The predominant production of *cis*-cyclo(l-Leu-l-Pro) (F13) and *cis*-cyclo(l-Phe-l-Pro) (F17) by *Lb. plantarum* LBP-K10 was used as a reference experiment.

*Ln. mesenteroides* LBP-K06, as one of the predominant (45.4%) *Leuconostoc* spp. among 205 isolates, has exhibited outstanding antibacterial performance on bacterial indicators, *E. coli* and *B. subtilis* (Supplementary Tables S1, S2). Inspired by the antibacterial activity of the tested material and to verify that CDPs are produced by *Ln. mesenteroides* LBP-K06, we examined HPLC retention times (*t*_R_) of every fraction that might directly relate to CDP peaks (Figure 1). The chromatographic separation of the culture filtrates displayed the particular *Leuconostoc* peaks distinguished by HPLC elution order. Interestingly, the two isolates showed very similar retention characteristics (Figure 1). However, the broad shoulder in the curved shape of the peak area from F13 to F15 of *Lb. plantarum* LBP-K10 differed from that of the completely sharp peak of N13 of *Ln. mesenteroides* LBP-K06.

**Figure 2:**
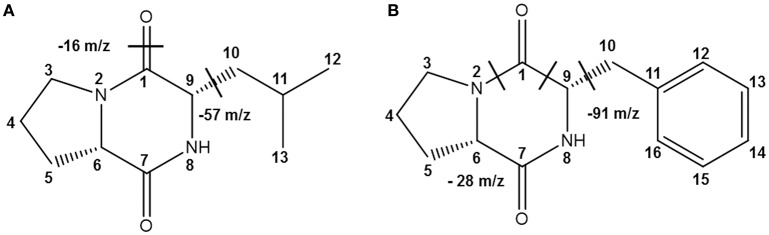
**The identified CDPs from fractions N113 and N15 from *Ln. mesenteroides* LBP-K06**. EI fragmentation patterns are indicated. EI-values of N13 and N15 and their fragmentation patterns have been assigned to be the proline-based **(A)** cyclo(Leu-Pro) (C_11_H_18_N_2_O_2_) and **(B)** cyclo(Phe-Pro) (C_14_H_16_N_2_O_2_), respectively.

Table 2 shows the antibacterial activity of each fraction of the cell-free filtrates from the growth of *Ln. mesenteroides* LBP-K06 in mMRS broth after primary HPLC fractionation on C18, strongly indicating a hydrophobic nature of antimicrobial compounds. As shown in the MIC assay, further separation of the fractions by HPLC and activity against *B. subtilis* and *E. coli* highlighted the two fractions, N13 and N15, of the 15 fractions collected. N13 and N15 were repeatedly subjected to semi-prep HPLC, developed with HClO_4_ extraction, and were recovered in amounts of 7.09 and 9.51 mg/L, respectively. We obtained the molecular ion [M+1]^+^ of fractions N13 and N15 at [M+1]^+^ 211 and 245, respectively. Strong evidence of identity was previously established by F13 and F17, including *cis*-cyclo(l-Leu-l-Pro) and *cis*-cyclo(l-Phe-l-Pro) (Kwak et al., 2013). As obtained from the distinctive chromatographic fractionation and GC-MS analysis, the EI values of N13 and N15 and their fragmentation patterns were assigned to be C_11_H_18_N_2_O_2_ and C_14_H_16_N_2_O_2_, the proline-based cyclo(Leu-Pro) and cyclo(Phe-Pro) (Figure 2, Table 3, and Supplementary Figure S1), respectively, coinciding with those produced by *Lb. plantarum* LBP-K10 (Kwak et al., 2013). These isolated CDPs showed significant antibacterial activity against Gram-positive (i.e., *B. subtilis* and *S. aureus*) and Gram-negative (i.e., *S. Typhimurium* and *E. coli*) reference bacteria and multidrug-resistant strains (i.e., *S. aureus*, 11471, and *S. Typhimurium*, 12219) (Table 4), indicating that the resulting MIC values of these compounds gave near identical status to those from *Lb. plantarum* LBP-K10 (data not shown).

**Table 2:** **Relative antibacterial activity of each fraction in the culture filtrate from *Ln. mesenteroides* LBP-K06**.

**Fraction number**	**N1**	**N2**	**N3**	**N4**	**N5**	**N6**	**N7**	**N8**	**N9**	**N10**	**N11**	**N12**	**N13**	**NF14**	**N15**
^1^ Antagonism test^a,^^*^	—	—	—	—	—	—	+	—	—	—	—	+	+++	—	+
^1^ Antagonism test^b,^^*^	—	—	—	—	—	—	+	—	—	—	—	+	+++	—	+
^2^ MIC^c,^^*^	—	—	—	—	—	—	—	—	—	—	—	49.5^3^	19.0	—	39.4
^2^ MIC^d,^^*^	—	—	—	—	—	—	—	—	—	—	—	50.1	16.4	—	37.6

1 *Symbol: +, < 15 mm; ++, < 22 mm; +++, > 22 mm (Indicator strains: B. subtilis^a^, E. coli^b^)*.

2 *MIC: Minimum inhibitory concentration (Indicator strains: B. subtilis^c^, E. coli^d^)*.

3 *mg/L*.

* The values represent the average ± SD of three independent experiments (^*^p < 0.05).

**Table 3:** **Mass analysis of *Ln. mesenteroides* LBP-K06 fractions by EI and CI using GC-MS**.

**Fractions**	**m/z of [M+1]^+^**	**m/z (%) of EI-MS**	**Predicted molecules**
N13 (22.5–23.5)^a^	211.0	54.0 (0.9), 55.0 (2.5), 56.0 (3.5), 57.0 (5.8), 58.0 (1.0), 69.0 (5.1), 70.0 (3.0), 71.0 (2.0), 72.0 (1.0),73.0 (1.8), 83.0 (1.3), 84.0 (4.3), 85.0 (3.6), 86.1 (10.5), 87.0 (0.9), 97.0 (1.1), 98.0 (1.9), 99.0 (4.3), 100.0 (1.3), 111.0 (1.4), 112.0 (1.3), 113.0 (23.4), 114.0 (1.5), 126.0 (1.4), 127.0 (1.6), 128.0 (100.0), 129.0 (6.6), 141.1 (5.2), 155.0 (1.4)	cyclo(Leu-Pro), C_11_H_18_N_2_O_2_
N15 (30.0–31.0)^a^	245.0	51.0 (2.0), 55.0 (4.2), 57.1 (4.5), 65.1 (4.8), 68.1 (5.4), 69.1 (6.7), 70.1 (44.1), 71.1 (4.9), 77.1 (4.3), 78.1 (2.2), 91.1 (40.3), 92.1 (10.8), 97.1 (2.2), 103.1 (5.1), 104.1 (6.1), 118.1 (2.8), 119.1 (2.3), 120.2 (15.4), 125.2 (100.0), 126.2 (7.9), 127.2 (3.5), 128.2 (2.8), 132.1 (3.5), 152.1 (3.7), 153.1 (48.4), 154.1 (4.4), 155.1 (2.7), 173.2 (2.9), 201.1 (3.6), 244.2 (88.3), 245.1 (15.4)	cyclo(Phe-Pro), C_14_H_16_N_2_O_2_

a *Retention time (min)*.

**Table 4:** **Antibacterial activity of N13 and N15 isolated from *Ln. mesenteroides* LBP-K06 filtrate**.

	**MIC (mg/L)^a^**	
	**N13, cyclo(Leu-Pro)**	**N15, cyclo(Phe-Pro)**
**INDICATOR STRAINS**		
**Gram-positive bacteria**		
*B. subtilis*	13.55	45.88
*S. aureus*	12.06	46.24
**Gram-negative bacteria**		
*S. Typhimurium*	12.98	40.03
*E. coli*	10.41^*^	35.68
**MULTIDRUG-RESISTANT STRAIN**		
**Gram-positive bacteria**		
*S. aureus* 11471^b^	17.28	46.22
**Gram-negative bacteria**		
*S. Typhimurium* 12219^c^	18.19^*^	43.7

a MIC: Minimum inhibitory concentration. The values represent the average ± SD (bars) of triplicate determinations as indicated (

* *p < 0.05)*.

b *Multidrug-resistant Gram-positive bacteria*.

c *Multidrug-resistant Gram-negative bacteria*.

Considering all of these data, we first found that *Ln. mesenteroides* secretes homologous antimicrobial CDPs and selected a potent antibacterial strain *Ln. mesenteroides* LBP-K06 as a principal fermenter to isolate CDPs in further study, based on the antibacterial activities of CDPs.

### The filtrate fractionation of kimchi fermented from chinese cabbage and from other plant materials

**Figure 3:**
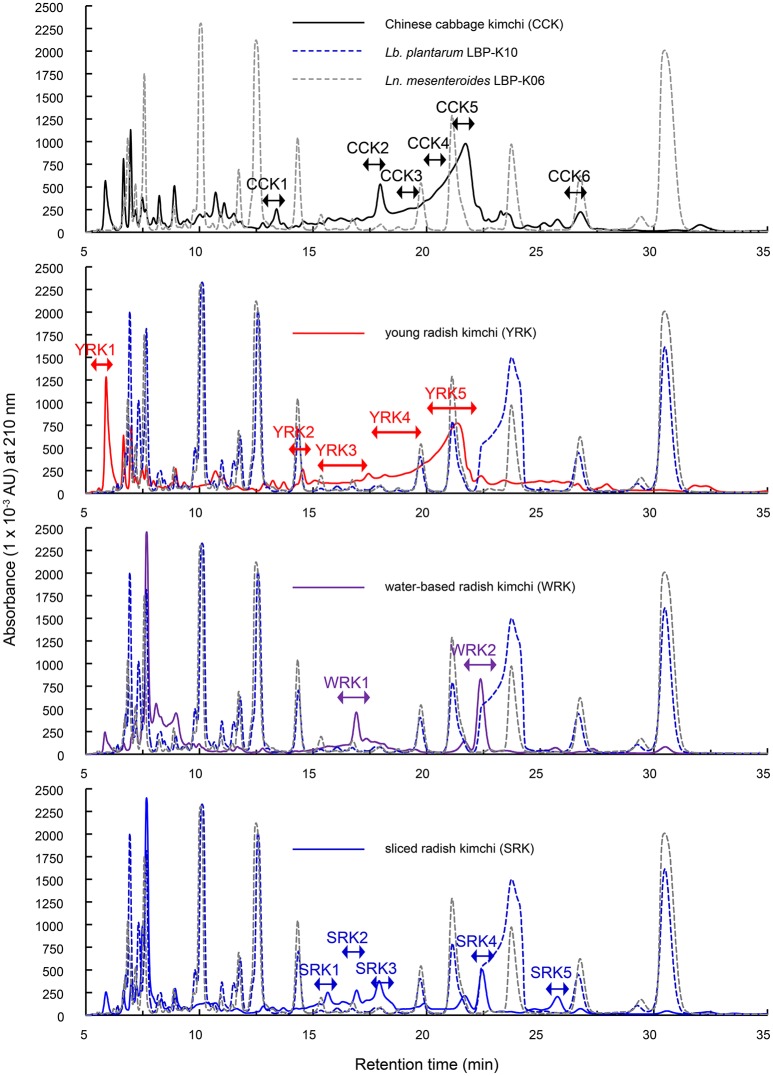
**Overall HPLC fractionated pattern of fermented kimchi products of plant origin using their whole filtrates as described in the materials and methods section**. The culture filtrates in the *Lb. plantarum* LBP-K10 and *Ln. mesenteroides* LBP-K06 were used as reference experiments, as indicated. A chromatographic analysis of all filtrates derived from LAB strains was performed at a wavelength of 210 nm. All experimental trials were repeated at least three times.

To further investigate the dynamic CDP production ability of *Ln. mesenteroides* LBP-K06 during kimchi fermentation, we preliminarily fractionated the filtrates to detect CDPs after spontaneously fermenting uninoculated CCK to the late kimchi fermentation stage (Figure 3) as proposed previously (Cheigh and Park, 1994). Even though we hypothesized that the observation of CDPs depends on achieving a particular fermented state by controlling the fermentation time of Chinese cabbage, other types of kimchi, including YRK, WRK, and SRK, also spontaneously fermented as a reference experiment along with bacterial culture filtrates. The HPLC fractionation was then subjected to GC-MS to validate purity of filtrate fractions, including CCK 1–6, YRK 1–5, WRK 1–2, and SRK 1–5. All kimchi filtrates typically seemed to have an increased amount of each fraction in common, particularly at retention times from ~15 to 25 min (Figure 3), consistent with *Lb. plantarum* LBP-K10 fractions from F8 to F15 as antimicrobial CDP-rich fractions (Kwak et al., 2013, 2014a).

However, a primary HPLC separation of filtrates demonstrated asymmetric and overlapping peaks absorbing at 210 nm (Figure 3) with retention times that did not coincide with those of any other CDP fraction from previously tested *Lactobacillus* CDPs (Kwak et al., 2013, 2014a). For example, although the naturally fermented WRK displayed a similar peak profile to that of SRK, and all peaks from SRK were slightly lower than those of WRK, these filtrates did not seem to contain any CDP peak, in contrast to the prediction from the bacterial-isolate culture filtrates such as N13 (F13) and N15 (F17) (Figures 1, 3). Additionally, no compound similar to CDP was observed in any of the fractions, including three types of undetectable YRK peaks (i.e., YRK1, YRK2, and YRK5) and all SRK peaks (i.e., SRK 1–5; Figure 3). These fractions were consecutively re-chromatographed on an ODS C18 column under a wide range of different isocratic mobile-phase composition combinations (i.e., 3, 5, 10, 15, and 20% methanol, 3, 5, and 10% acetonitrile, and 67–94% HPLC grade water). Despite elaborate experimental trials to obtain pure components through the recursive HClO_4_ extraction and further individual fraction separations, it was difficult to identify peak-area normalization for quantitative impurity content determination. We also failed to verify the relative retention peak area proportion relevant to the fraction cyclo(Phe-Pro) in all types of filtrates in contrast to the isolated bacterial culture filtrates (Figure 1). Nevertheless, CCK showed a completely different fractionated profile from any other kimchi and bacterial isolates (Figure 3), indicating that the different chromatographic profile of CCK compared to other types of materials might be due to kimchi species-specific characteristics during fermentation. Additionally, in non-starter kimchi, we observed no molecular ions in their poor EI mass spectra, and also fragment ions did not clearly appear from uncertain molecular ions and high-resolution mass measurements (data not shown).

Specifically, because several peaks in radish kimchi fused together, depending on the degree of overlap, the overlapping peaks that might impact each other were more separated by using different isocratic mobile-phase compositions at various pHs to distinguish primary peaks before making accurate estimates of any parameter. Despite efforts to isolate single peaks from complex chromatograms (Figure 3), we could not isolate even a single compound in all types of kimchi, despite controlling experimental parameters of elution volume at peak maximum and peak height.

### Identification of the increased fractions of filtrates from CCK produced with *Ln. mesenteroides* LBP-K06 starter cultures by fermentation time

**Figure 4:**
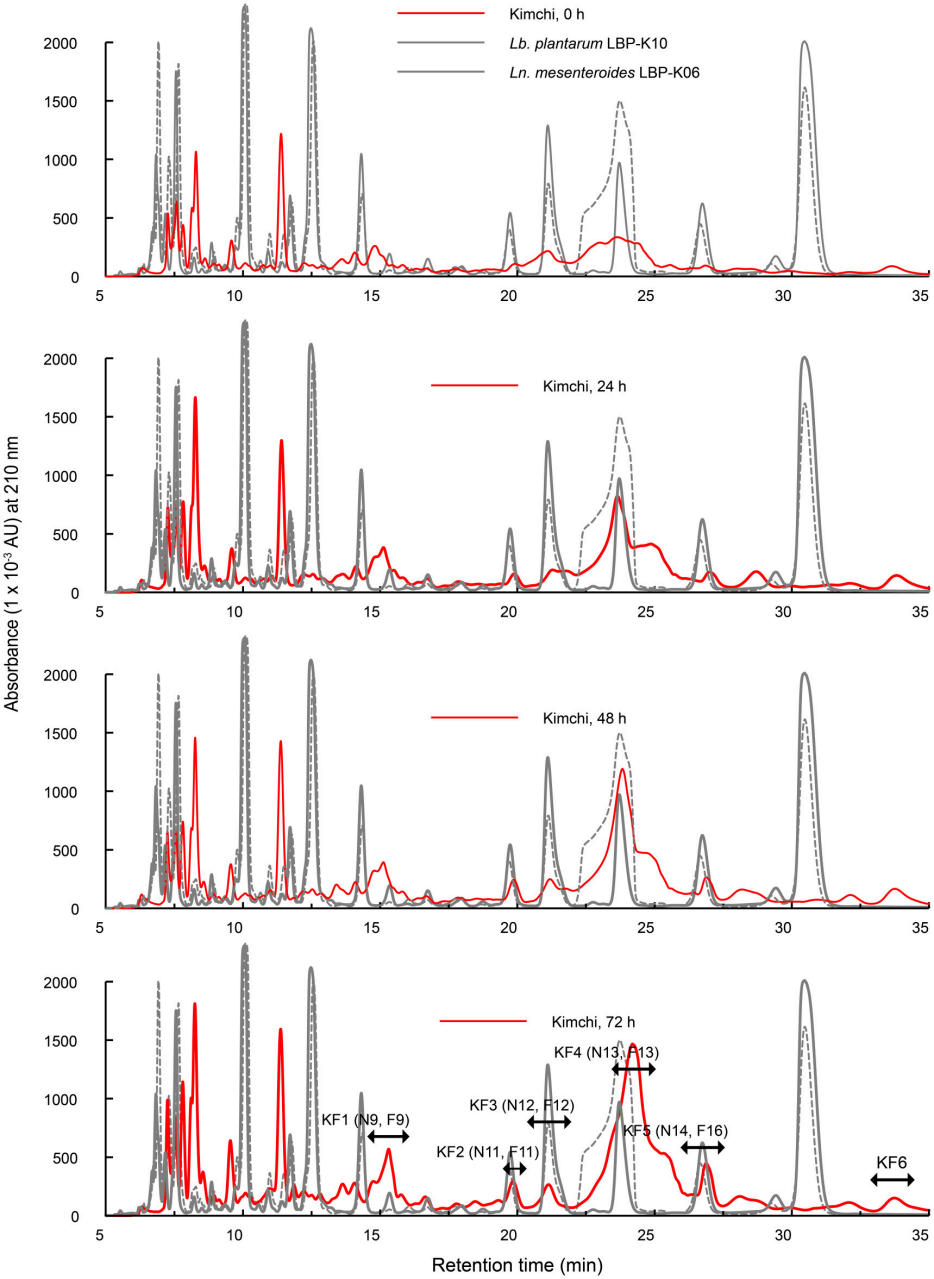
**Comparisons of the chromatograms of kimchi filtrates obtained from Chinese cabbage kimchi inoculated with *Ln. mesenteroides* LBP-K06**. Chinese cabbage kimchi was further incubated for 72 h at 25°C after the initial kimchi-fermentation stage. A chromatographic separation of kimchi filtrates was performed at a wavelength of 210 nm. Each experiment was performed at least three times independently.

Considering the experimentally confirmed unique peak shape and distribution derived from chromatographic resolution characteristics by fermentation source (Figure 3), the use of defined starter strains in fermented foods might be pivotal in maintaining starter predominance to control fermentation by microbial growth. We thus had to use Chinese cabbage fermentation filtrates obtained from starter kimchi inoculated with *Ln. mesenteroides* LBP-K06 in the early kimchi fermentation stage. Moreover, we hypothesized additional fermentation to monitor whether significant peak-pattern change would occur by rapid fermentation at 25°C for 72 h to the middle kimchi fermentation stage. In contrast to non-starter kimchi, we observed distinct CDP-like peaks designated KF1 to KF6 and their content changed in chromatograms by fermentation time (Figure 4). Surprisingly, HPLC peaks constructed with the retention time–peak area data matrices ranging from 15 to 25 min were almost consistent with those of bacterial culture filtrates, including F9 (KF1), F11 (KF2), F12 (KF3, N12), F13 (N13, KF4), and F16 (N14, KF5; Figure 4). We then structurally investigated the isolated fractions by GC-MS after conversion to lyophilized compounds. Molecular ion [M+1]^+^ of fractions KF1 and KF2 were obtained at [M+1]^+^ 185 and 261, respectively. Compared to other fractions, the dramatically more increased fraction KF4 was predicted to be cyclo(Leu-Pro) by CI [M+1]^+^, 211, similar to *cis*-cyclo(l-Leu-l-Pro) in our previous work (Table 5) (Kwak et al., 2013).

**Table 5:** **Mass analysis of fractions of kimchi filtrates from fermented kimchi inoculated with *Ln. mesenteroides* LBP-K06 by EI and CI using GC-MS**.

**Fractions**	**m/z of [M+1]^+^**	**m/z (%) of EI-MS**	**Predicted molecules**
KF1 (15.0–17.5)^a^	185.0	55.1 (7.9), 57.1 (6.5), 70.1 (13.6), 71.1 (3.4), 86.1 (20.1), 91.1 (0.5), 98.1 (7.2), 99.1 (9.9), 113.1 (13.6), 127.1 (1.0), 128.1 (100.0), 129.1 (5.6), 141.2 (9.9), 169.2 (0.5)	cyclo(Ser-Pro), C_8_H_12_N_2_O_3_
KF2 (19.0–20.5)^a^	261.0	55.0 (11.2), 56.1 (4.3), 68.1 (10.8), 70.1 (4.8), 86.1 (32.8), 91.1 (77.1), 92.1 (13.9), 99.1 (1.3), 113.1 (4.9), 120.1 (21.6), 128.1 (49.3), 131.1 (4.5), 141.1 (81.8), 142.1 (2.7), 169.1 (86.8), 170.1 (5.7), 258.2 (1.3), 260.2 (100.0), 261.2 (16.9)	cyclo(Tyr-Pro), C_14_H_16_N_2_O_3_
KF4 (21.5–23.5)^a^	211.0	55.0 (6.3), 68.1 (5.4), 69.1 (7.3), 70.1 (54.4), 86.1 (20.4), 96.1 (4.2), 124.1 (8.1), 125.1 (9.4), 139.1 (5.9), 154.1 (100.0), 155.1 (8.8), 167.1 (5.5)	cyclo(Leu-Pro), C_11_H_18_N_2_O_2_

a *Retention time (min)*.

**Figure 5:**
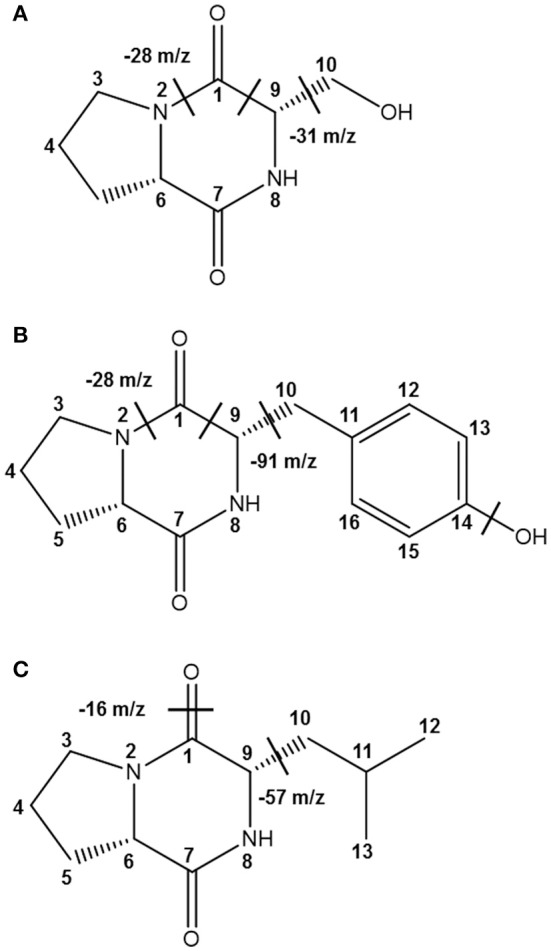
**The identified CDPs from fractions KF1, KF2, and KF4 from Chinese cabbage kimchi inoculated with *Ln. mesenteroides* LBP-K06 starter cultures**. EI fragmentation patterns are indicated. Structural units separated by chemical bonds are divided by dashed lines. Mass fragmentation pattern under EI of these compounds have been revealed to be **(A)** cyclo(Ser-Pro) (C_8_H_12_N_2_O_3_), **(B)** cyclo(Tyr-Pro) (C_14_H_16_N_2_O_3_), and **(C)** cyclo(Leu-Pro) (C_11_H_18_N_2_O_2_), respectively.

Together with the mass fragmentation pattern under EI, we determined these compounds to be C_8_H_12_N_2_O_3_, C_14_H_16_N_2_O_3_, and C_11_H_18_N_2_O_2_, corresponding to cyclo(Ser-Pro), cyclo(Tyr-Pro), and cyclo(Leu-Pro), respectively (Table 5, Figure 5), and Supplementary Figure S2). However, fraction KF5, which showed accordance with N14 and F16, increased at 24 h during further rapid fermentation, showing the same retention characteristics (Figure 4). The established molecular ion peak was not observed in this fraction. Although predicted from chromatographic peak similarity between N12 and F12 in bacterial isolates (Figure 1), fraction KF3 showed unchanging peak-to-peak amplitudes and line widths for 72 h and did not contain any compound identical to the non-starter CCK (Figure 3). Therefore, we confirmed changes in the identified CDP contents in the starter kimchi by fermentation time (Table 6), as well as different antibacterial activity of the starter kimchi fractions against bacterial indicators following primary separation of filtrates by HPLC (Supplementary Table S3).

**Table 6:** **Changes in total amounts of CDPs in kimchi filtrates measured by semi-prep HPLC followed by MC extraction**.

**Identified compound**	**Total amount of the culture filtrates in CFs and kimchi filtrates from *Ln. mesenteroides* LBP-K06**				
	**culture filtrate**	**kimchi filtrate, 0 h**	**kimchi filtrate, 24 h**	**kimchi filtrate, 48 h**	**kimchi filtrate, 72 h**
	**mg/L^a^**	**mg/L^a^**	**mg/L^a^**	**mg/L^a^**	**mg/L^a^**
KF1, cyclo(Ser-Pro)		0.75 ± 0.19	1.52 ± 0.23	1.99 ± 0.36	2.67 ± 0.31^*^
KF2, cyclo(Tyr-Pro)		0.88 ± 0.14	1.48 ± 0.13^*^	2.16 ± 0.17	2.96 ± 0.33
KF4, cyclo(Leu-Pro)		1.69 ± 0.21	2.24 ± 0.33^*^	3.85 ± 0.78	8.42 ± 0.74
N13, cyclo(Leu-Pro)	7.09 ± 0.39	—	—	—	—
N15, cyclo(Phe-Pro)	9.51 ± 0.77	—	—	—	—

a Data presented as means with standard deviation (SD). Asterisks represent statistically significant values (

* *p < 0.05)*.

### Antibacterial activity comparison between isolated CDPs from bacterial culture filtrates and from CCK produced with *Ln. mesenteroides* LBP-K06 starter cultures

Next, the antibacterial activities of the identified CDP fractions from the starter kimchi were determined through MIC dilution assays against bacterial indicators and multidrug-resistant bacteria. We compared the MIC values of each fraction to determine whether the antimicrobial activities of starter-kimchi CDPs might be consistent with those of *Ln. mesenteroides* LBP-K06 culture filtrates (Tables 4, 7). The CDP fractions from the *Leuconostoc* culture filtrates did have significant antibacterial activity against reference strains and multidrug-resistant bacteria (Tables 4). We found that the kimchi-filtrate CDP fractions had an MIC of ~11.0–14.0 and 17.0–19.5 mg/L for cyclo(Leu-Pro) against bacterial indicators and multidrug-resistant bacteria, respectively, corresponding to 10.0–14.0 mg/L for fraction N13 cyclo(Leu-Pro) from bacterial culture filtrates.

**Table 7:** **Antibacterial activity of identified CDPs from starter kimchi against Gram-positive and Gram-negative bacterial indicators and multidrug-resistant bacteria**.

	**(mg/L)^a^^,^^d^**		
	**KF1 cyclo(Ser-Pro)**	**KF2 cyclo(Tyr-Pro)**	**KF4 cyclo(Leu-Pro)**
**INDICATOR STRAINS**			
**Gram-Positive Bacteria**			
*B. subtilis*	—	26.78	13.96
*S. aureus*	—	31.55	11.45
**Gram-Negative Bacteria**			
*S. Typhimurium*	—	26.1	11.78
*E. coli*	—	22.32	11.3
**MULTIDRUG-RESISTANT STRAINS**			
**Gram-Positive Bacteria**			
*S. aureus* 11471^b^	—	29.71	17.98
**Gram-Negative Bacteria**			
*S. Typhimurium* 12219^c^	—	30.64	19.33

a *MIC: Minimum inhibitory concentration*.

b *Multidrug-resistant Gram-positive bacteria*.

c *Multidrug-resistant Gram-negative bacteria*.

d The values represent the average ± SD (bars) of triplicate determinations as indicated (^*^p < 0.05).

Specifically, for the Gram-positive and Gram-negative indicator strains, including *B. subtilis, S. aureus, S. Typhimurium*, and *E. coli*, the total growth-inhibition MIC were 13.96, 11.45, 11.78, and 11.3 mg/L, similar to the MIC for cyclo(Leu-Pro) from the *Leuconostoc* filtrate fraction N13, 13.55, 12.06, 12.98, and 10.41 mg/L (Table 4). Additionally, the MIC of 17.98 and 19.33 mg/L for cyclo(Leu-Pro) against multidrug-resistant bacteria showed a similar MIC pattern to a bacterial isolate, 17.28 and 18.19 mg/L. However, the MIC of cyclo(Leu-Pro) against the multidrug-resistant Gram-positive and Gram-negative bacteria were significantly higher, approximately 1.51–1.86 fold, than those against the Gram-positive and Gram-negative indicators (Tables 4, 7). The antibacterial activity of cyclo(Tyr-Pro) against bacterial reference strains was lower than that of cyclo(Leu-Pro), showing that Gram-positive or Gram-negative bacterial status was confirmed by the cyclo(Tyr-Pro) MIC of 26.1 and 11.3 mg/L or above. However, the isolated cyclo(Ser-Pro) was not bioactive, as determined by MIC using bacterial indicator strains.

### The absence of CDPs in other fermented foods of plant or animal origin

To support our hypothesis about CDP production in the CCK starter in contrast to spontaneously fermented kimchi (Figure 4, Tables 5, 6), several types of non-starter fermented products, plant and animal, were analyzed for CDPs and their analogs. We screened the filtrates derived from fermented plant materials—including soybean paste, *B. subtilis* nattō, soy sauce, and fast-fermented bean paste—for CDPs (Supplementary Figure S3). We tried to verify fractions from soy sauce and fast-fermented bean paste filtrates (i.e., SS 1-6 and BP 1-2). However, the resulting peaks in HPLC could not be used to separate and collect fractions in all tested fermented products because the HPLC profiles of the corresponding fractions displayed severely overlapping peaks and further fractionated peaks also showed increasing retention volume with unseparated peaks. Thus, the HPLC could not separate pure compounds and also no activity was observed when tested against bacterial indicators. Moreover, the EI/CI values could not be used to calculate the exact mass of molecules as they displayed very poor ionization patterns with few signals in these fermentation filtrates (data not shown). This phenomenon was likely due to the naturally fermented kimchi (Figure 3), indicating both the limitation of requirements for amount of samples and the decisive quantification difficulty of all analytes due to the overlapping peaks from poor chromatographic fractionation. This result also suggests that overlapping peaks might consist of various types of inseparable compounds. In the case of fermented materials of animal origin, two types of Korean traditional pickled or salted shrimp and clams also conveyed no peaks containing CDPs (Supplementary Figure S4), and the subsequent GC-MS analysis revealed nothing of value. Interestingly, a predominant strain in pickled or salted shrimp was *Staphylococcus equorum* using 16S rRNA sequencing (Supplementary Figure S5) among isolates, coinciding with previous investigation (Jeong et al., 2014). Therefore, we concluded that *Ln. mesenteroides* could synthesize CDPs in the culture medium and in starter kimchi, but not in non-starter fermented materials. Furthermore, non-LAB species in some types of traditional Korean fermented foods did not seem to be involved in producing CDPs, although many types of Gram-positive or -negative bacteria were elucidated to produce and excrete CDPs, as previously reported (Kwon et al., 2000; Rhee, 2002; Yan et al., 2004; Lind et al., 2007; Huang et al., 2010, 2014).

## Discussion

Based on our previous findings (Kwak et al., 2013, 2014a), we used bacterial filtrates from the supernatant and freeze-dried powder of all kinds of kimchi, readily available for CDP purification. This strategy prominently comes from repeated methodological development applying 10-fold HClO_4_ extraction with remarkable selectivity for CDP isolation and several time change of mobile phase, strongly rendering CDPs sufficiently pure to be analyzed and confirmed by GC-MS for EI/CI mass spectrometry as a single compound without any other peptidyl or non-peptidyl compounds. By using this CDP characterization model, we exploited specific-strain starter systems because use of filtrates from non-starter kimchi have been proven to have limitations for collecting any CDP or single fraction directly due to poor chromatographic separation (Figure 3). This result strongly suggests the possible interference of a hydrophobic nature by poor chromatographic-driven polar-component content changes commonly observed in foodstuffs (Young, 2016), crucially arising from secondary metabolite profile alteration during spontaneous fermentation by necessarily complex heterofermentative microflora (Jung et al., 2012). This phenomenon can be reliably and meaningfully evidenced by secondary byproduct changes of starter and non-starter kimchi affected by the controlled microflora (Jung et al., 2012, 2014; Jeong et al., 2013), along with more poor chromatographic retention behaviors of the analytes caused by polar-compound increases resulting from metabolite changes of foodstuffs (Young, 2016).

Other studies also support our current work regarding starter and non-starter kimchi, indicating that *Ln. mesenteroides* starter normally maintains its predominance ~88% during fermentation (Eom et al., 2008) as an absolute dominant over *Lb. plantarum* and other microflora during the early kimchi fermentation stage (Jung et al., 2012). To prevent an influx of other kimchi microflora (e.g., *Leuconostoc* spp., *Lactobacillus* spp., and *Weissella* spp.) governing fermentation rate, microbial community, and extra metabolite production (Lee et al., 2008; Chang and Chang, 2010), we thereby modeled the kimchi starter using Chinese cabbage, which has been widely used in Korea for making ordinary kimchi (Jung et al., 2011, 2012, 2014), inoculated with *Ln. mesenteroides* LBP-K06 as a strong candidate for antimicrobial CDP-biosynthesizing isolate (Figure 4).

Underlying principles of this fermentation control by starter cultures primarily come from the evaluation of poor chromatographic separation of non-starter cultures with no detection of CDP peaks in our experiments. Simultaneously, different from a naturally fermented kimchi for dairy use, our factorial design-based study controlling fermentation process parameters (Panda et al., 2007) including temperature, pH, time, and inoculum amount, has been employed as a close-ended system in every screening round when and whether testing the primary CDP detection according to the type of raw materials and thereafter with the secondary use of starter cultures, to efficiently produce/characterize bioactive CDPs. The effect of designed fermentation strategies herein remarkably enhanced the yield of detectable CDPs that are most importantly dependent on parameters using the starter strain *Ln. mesenteroides* LBP-K06 (Figure 3) and the specific raw material Chinese cabbage (Figure 4). These two-type parameter-controlled fermentations accompanying fermentation time-course modifications adjusted by fermentation temperature experimentally facilitated the monitoring of different HPLC chromatographic separation patterns, showing and not showing distinctly changing CDP peaks between the controlled and the spontaneously fermented products.

Although the production of kimchi CDPs in a close-ended system is evidently proven by the microbial community from starter cultures used as a predominant fermentation parameter, the entire byproduct contents resulting from metabolite changes that undergo fermentation also seem to closely align with the type of kimchi material from our results. This assumption lies in that a different dietary composition and ratio in raw materials of plant (or animal) origin may drive different fermentation metabolite production coupled with defined starter-culture regulation. Results of the content analysis by a previous study (Jung et al., 2012) convincingly supported our hypothesis, suggesting the significant quantitative difference of CCK and YRK carbon sources, such as glucose and fructose. The significant different levels of carbon source profiles in raw materials before fermentation maintain constantly until the middle stage of fermentation and thereby fundamentally can influence LAB metabolism in the production of various fermentation products. Specifically, free sugars in Chinese cabbage are relatively higher than those in radish commonly found in raw materials and fermented kimchi.

Interestingly, other evidence of differences in food-composition content of raw materials affecting metabolite production during fermentation is explained by free AA concentrations in raw vegetables (Kim et al., 2009). As illustrated in the different content of carbon sources between Chinese cabbage and radish, the verified content of various AAs in these raw materials also show an almost similar pattern to carbon sources. The corresponding content of all kinds of AAs, such as branched AAs, sulfur-containing AAs (i.e., methionine and cysteine), and aromatic AAs (i.e., phenylealanine and tyrosine), represent the relative quantitative difference among the raw vegetables. Thus, the relatively lower level of nutrient availability driven by the raw vegetables seems to be responsible for affecting the metabolite profile in fermented products, coinciding with a non-starter WRK study showing remarkably lowered amounts of metabolites (Jeong et al., 2013), including AAs, organic acids, glucose, and fructose, in contrast to those in starter and non-starter CCK and YRK at the same fermentation stage (Jung et al., 2012). Similarly, our HPLC data and further CDP purification study from several types of radish kimchi (SRK, WRK, and YRK) also do not correlate with CDP production, presumably due to the relatively and remarkably lower levels of AAs and carbon sources of raw materials than those of Chinese cabbage. These phenomena imply that kimchi metabolites are important dietary components and their composition might be partly applied to predicting, estimating, or evaluating CDP-producing behaviors along with kimchi tastes or flavors. Hence, changes in metabolite compositions, including organic acids (i.e., lactic and acetic acids) and flavoring compounds (i.e., mannitol and amino acids) (Ha et al., 1989), can give a partial possibility of CDP action during fermentation according to type of kimchi material. Therefore, for example, we suggest here Chinese cabbage with starter *Ln. mesenteroides* to make CDP-rich kimchi to meet CDP functions. Our data convincingly demonstrate, for the first time, CDP production in the controlled fermented cabbage—under the control of specific fermentation-process parameters, including starter dominance, time-course modification, and temperature adjustment—concomitantly coupled with strong CDP selectivity by using a two-consecutive purification strategy.

Although negligible differences emerged during the entire fermentation period in the production of organic acids and mannitol in fermented vegetables (Jung et al., 2012), which is a naturally occurring, non-carcinogenic, and diabetic polyol at high levels in kimchi, the use of *Ln. mesenteroides* as a starter culture inspires commercial production of fermented kimchi (Grobben et al., 2001). In the present study, 85.7% of fermented kimchi by obligately heterofermentative microflora is occupied by Chinese cabbage (Ji et al., 2009). This commercial trend is also thought to facilitate monitoring and assessment of starter *Leuconostoc*-driven CDPs.

In contrast, in the case of culture filtrates of *Lactobacillus* and *Leuconostoc* cells, CDPs significantly showed the time point with the highest total amount at 72 h simultaneously with a decline in cell numbers (Kwak et al., 2013) (Figure 1). Similar relationships are observed in *agr*-mediated dual-channel quorum-sensing signaling engaged in CDP production of *Lb. reuteri* RC-14 cyclo(l-Phe-l-Pro) and cyclo(l-Tyr-l-Pro) (Li et al., 2011). These growths of *Lactobacillus* or *Leuconostoc* species in culture media have been commonly recognized to require complex nitrogen sources (Amoroso et al., 1993; Elli et al., 1999). Growth behaviors entirely derived from complex medium in laboratory experiments seem to be very attractive for use in fermentation standardization, but completely different from Chinese cabbage or other raw-material fermentation when seen as part of the AA use pattern, whether used in growth in media or raw materials of plant or animal origin. Chinese cabbage normally contains only 663 mg of total AA in 100 g of raw vegetables (Ji et al., 2009) completely different from rich MRS medium. Additionally, *Leuconostoc* strains used in the cheese-making process show that AA use is limited to branched-chain AAs in the case of milk, because l-leucine and l-valine commonly act as competitive inhibitors occupied by branched AA transport systems for the uptake of AAs in *Ln. mesenteroides* (Mayshak et al., 1966; Winters et al., 1991; Foucaud et al., 2001). Hence, these properties of having species-specific metabolism presumably affecting AA concentrations and consecutively other metabolite changes are also thought to align with aspects of a key feature of CDP production in raw material fermentation affected by fermentation control parameters.

Specifically, CDPs containing 2, 5-diketopiperazines come from their rigid backbone that can mimic preferential peptide conformations and contain highly constrained AAs essentially resulting from the double condensation of two α-AAs (Ciarkowski, 1984). Because starter or non-starter ripening flora uses AAs to a greater extent as part of their primary metabolic activities in fermenting kimchi, AA consumption influenced by CDP biosynthesis seems to be in line with a previous finding of gradually decreased AA content after the middle fermentation stage (Jeong et al., 2013). As evidence for this hypothesis, proline-based CDP fractions, including KF1, KF2, and KF4, showed remarkably higher content proportional to fermentation time compared to those of starter kimchi at the early fermentation period (0 h) (Figure 4 and Table 6). Moreover, the absence of CDP, especially cyclo(Phe-Pro), in the non-starter kimchi (Figure 3) and in other spontaneously fermented products (Supplementary Figures S3, S4), in contrast to the presence of cyclo(Ser-Pro), cyclo(Tyr-Pro), and cyclo(Leu-Pro) in the controlled fermented cabbage (Table 4), also coincidentally correspond to previously established experiments on the remarkably lower content of specific AAs, including tyrosine, histidine, threonine, alanine, valine, phenylalanine, isoleucine, leucine, and methionine, in spontaneously fermented cabbage, compared to controlled fermented cabbage using *Ln. mesenteroides* NCIM 2073 as a starter after the middle kimchi fermentation stage (Jagannath et al., 2012). Additionally, finding cyclo(Tyr-Pro) containing one type of aromatic AA, tyrosine (Table 5), also seems to be caused by the use of the defined starter *Ln. mesenteroides* LBP-K06 because hydroxy phenyllactic acid produced from tyrosine has an antimicrobial spectrum as phenyllactic acid, particularly in starter kimchi (Crowleya et al., 2013; Naz et al., 2013). These results strongly imply that spontaneously fermented cabbage is likely to have a varied microflora, as seen in the very different chromatographic separation pattern and CDP production compared to starter kimchi (Figures 3, 4).

Considering the antibacterial activity of cyclo(Leu-Pro) against multidrug-resistant *S. aureus* 11471 and *S. Typhimurium* 12219, we observed MIC values (Table 7) similar to 17.28 and 18.19 mg/L for cyclo(l-Leu-l-Pro) from *Ln. mesenteroides* LBP-K06 culture filtrates. In the case of cyclo(Tyr-Pro), its active concentration against bacterial strains seems to be very similar to the MIC of 31.25 mg/L from previous investigations of *Streptomyces* sp. strain 22-4 cyclo(l-Pro-l-Tyr) and cyclo(d-Pro-l-Tyr), which displayed antibacterial activity against *Xanthomonas axonopodis* pv. Citri and *Ralstonia solanacearum* (Wattana-Amorn et al., 2015), although different pathogenic strains were used to test antimicrobial activity. These results also coincide with previous reports that cyclo(Leu-Pro) (Yang et al., 2011), cyclo(Tyr-Pro) (Kwak et al., 2014a), and cyclo(Phe-Pro) have antibacterial or antifungal activities (Ström et al., 2002). Additionally, maturation of gastrointestinal cells was enhanced by synthetic cyclo(Phe-Pro) and cyclo(Tyr-Pro) (Graz et al., 1999). Interestingly, the antimicrobial CDP fractions from kimchi filtrates (Table 5) from *Ln. mesenteroides* LBP-K06 and *Lb. plantarum* LBP-K10 were mainly found in the latter part of HPLC chromatogram from ~15 to 32 min from N9 (F9) to N15 (F17).

Additionally, non-antimicrobial fractions of *Leuconostoc* and kimchi filtrate were displayed in the forepart of the chromatograms (Table 2). These results coincide with our preliminary experiments showing that the significantly higher amounts of CDPs only from F11 to F15 were produced by l-proline-supplemented *Lactobacillus* cells in the presence of D-glucose under buffer conditions; glucose-depleted or -supplemented buffer conditions made no change in the amount of other fractions irrelevant to the presence or absence of L-proline (data not shown). Similarly, the relative content of cyclo(Leu-Pro) and cyclo(Tyr-Pro) from starter kimchi were significantly more affected than other fractions by fermentation time (Figure 4 and Table 5), suggesting that fermenting-condition optimization (ripening time and temperature) could change CDP content in kimchi. Also, despite differences in the amount of antimicrobial substances among isolates (Table 6), *Ln. mesenteroides* excreted CDPs that acted as bioactive mediators, found in common with specific kimchi types. Therefore, the time-dependent fermentation control strategy for screening CDPs in CCK suggests that *Ln. mesenteroides* LBP-K06 inoculation as a predominant starter might facilitate detection of CDPs, whereas food fermentation, usually relying on naturally inoculated (inherent) microbial flora, resulted in variable and uncontrolled product quality. This result also corresponds with the result that naturally fermented materials might be insufficient to evaluate the starter and its predominance in such complex and mixed-strain microbial variety in foods (Giraffa and Rossetti, 2004).

## Conclusion

Our current study aimed to demonstrate CDPs in Korean fermented products with antimicrobial activity against pathogenic microbes for the first time. Our results focused on controlled Chinese-cabbage fermentation with a single-strain starter culture, presumably affecting the growth of LAB and their metabolites. This hypothesis was reflected in the significant proline-based CDP production of starter kimchi in contrast to that of other fermented products. Moreover, CDP production during kimchi fermentation was observed to be different from culture filtrates of *Ln. mesenteroides* LBP-K06. The unique chromatographic profile of Chinese cabbage filtrates of starter kimchi inoculated with *Ln. mesenteroides* LBP-K06 can be used to isolate unknown compounds using other fermented materials in further study.

This is the first report showing that antibiotic CDPs in CCK filtrates might be applied for antimicrobial preservation and other purposes. Based on experimental clues from MIC determination, we demonstrated the active concentration of each CDP in kimchi filtrates to be similar to that of CDPs in previously established bacterial filtrate fractions. Our experiments confirm beneficial substances in kimchi. These findings can provide a framework for future research or industry regarding the fermenting process or of making kimchi. Additionally, antibiotic CDPs in these fermented products might provide the capability to assess the antibiotic effects of CDPs and other possible applications.

## Author contributions

MK, RL, AK, and SK designed the research. MK, RL, and AK performed the research. MK, RL, AK, and SK analyzed the data. MK and RL contributed new reagents/analytic tools. MK, RL, and SK wrote the manuscript with significant input from the others.

### Conflict of interest statement

The authors declare that the research was conducted in the absence of any commercial or financial relationships that could be construed as a potential conflict of interest.

## Abbreviations

AA: amino acid
CCK: Chinese cabbage kimchi
CDP: cyclic dipeptide
CI: chemical ionization
EI: electron ionization
GC-MS: gas chromatography–mass spectrometry
HPLC: high-performance liquid chromatography
MIC: minimum inhibitory concentration
MRS: de Man, Rogosa and Sharpe agar
ODS: octadecyl silica
SRK: sliced radish kimchi
WRK: water-based radish kimchi
LAB: lactic acid bacteria
YRK: young radish kimchi.
