# Supplementary material for: Proline-Based Cyclic Dipeptides from Korean Fermented Vegetable Kimchi and from Leuconostoc mesenteroides LBP-K06 Have Activities against Multidrug-Resistant Bacteria
Source: Front Microbiol. 2017 May 2;8:761. doi: 10.3389/fmicb.2017.00761 (PMC5411444; doi:10.3389/fmicb.2017.00761)
Supplement: Supplementary file 1 [file DataSheet1.PDF]

[Frontiers in Microbiology]
Manuscript ID: 214633

Supplementary material:

Proline-based cyclic dipeptides from Korean fermented vegetable kimchi and from *Leuconostoc mesenteroides* LBP-K06 have activities against multidrug-resistant bacteria

Rui Liu, Andrew HyoungJin Kim[1], Min-Kyu Kwak, Sa-Ouk Kang**

Laboratory of Biophysics, School of Biological Sciences, and Institute of Microbiology, Seoul National University, Seoul 151-742, Republic of Korea

**** Correspondence:***

Min-Kyu Kwak, Laboratory of Biophysics, School of Biological Sciences, and Institute of Microbiology, Seoul National University, Seoul 151-742, Republic of Korea. Tel.: +82 2 880 6703; fax: +82 2 888 4911. genie6@snu.ac.kr

Sa-Ouk Kang, Laboratory of Biophysics, School of Biological Sciences, and Institute of Microbiology, Seoul National University, Seoul 151-742, Republic of Korea. Tel.: +82 2 880 6703; fax: +82 2 888 4911. kangsaou@snu.ac.kr

Running title: *Quantification of cyclic dipeptides in fermented foods*

[1] ***Present address:*** *Department of Clinical Pharmacology and Therapeutics, Seoul National University. College of Medicine and Hospital, Seoul 110-744, Republic of Korea*

Supplementary Figures

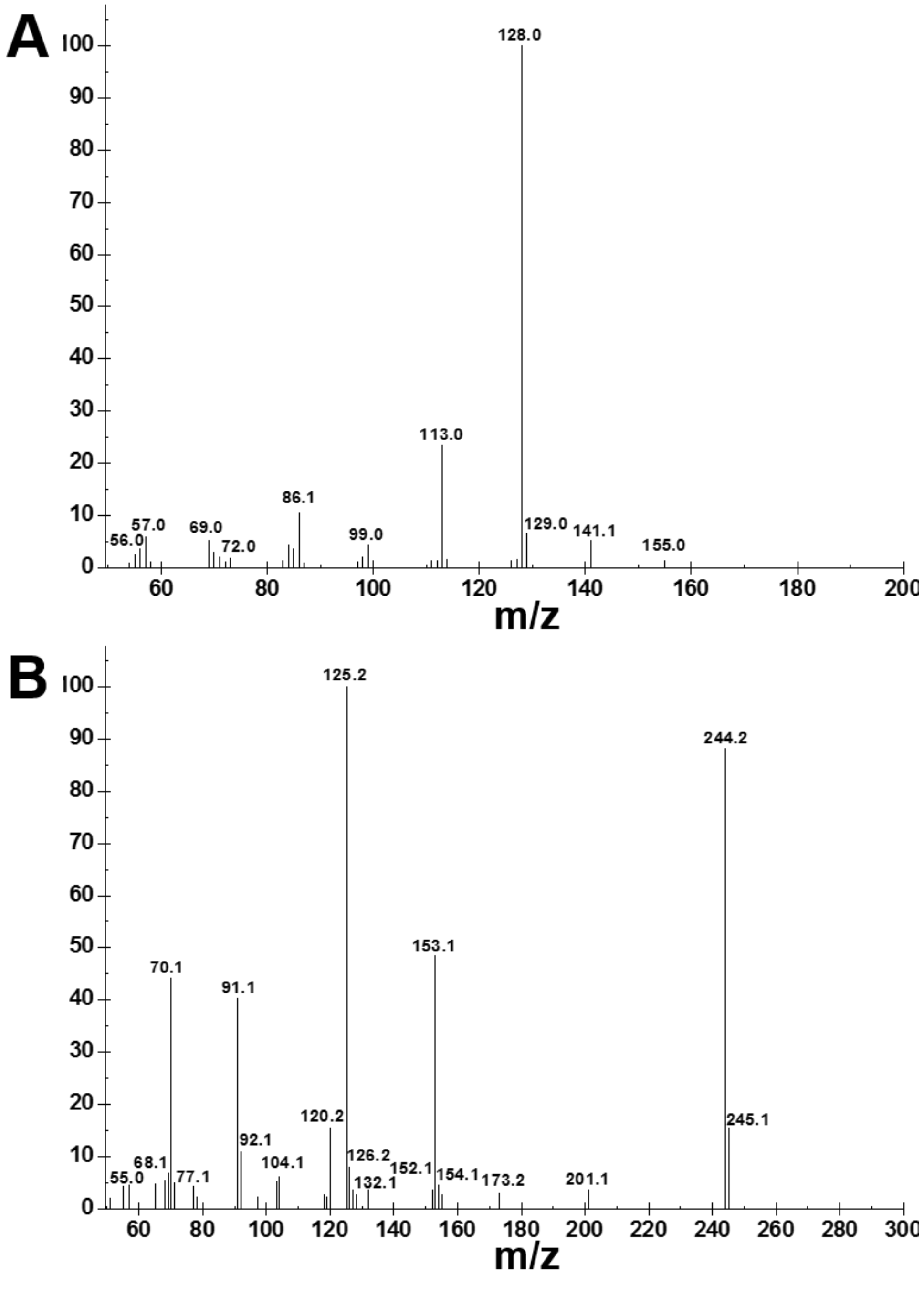

SUPPLEMENTARY FIGURE S1| Structural analysis of two fractions from *Ln. mesenteroides* LBP-K06. (A) and (B) corresponding to N13 and N15 were obtained by electron impact using GC-MS. The EI and CI values of each fraction were suggested in **Table 3**. The fragmentation patterns were assigned to be (A) $C_{11}H_{18}N_2O_2$ and (B) $C_{14}H_{16}N_2O_2$ as the proline-based cyclo(Leu-Pro) and cyclo(Phe-Pro), respectively.

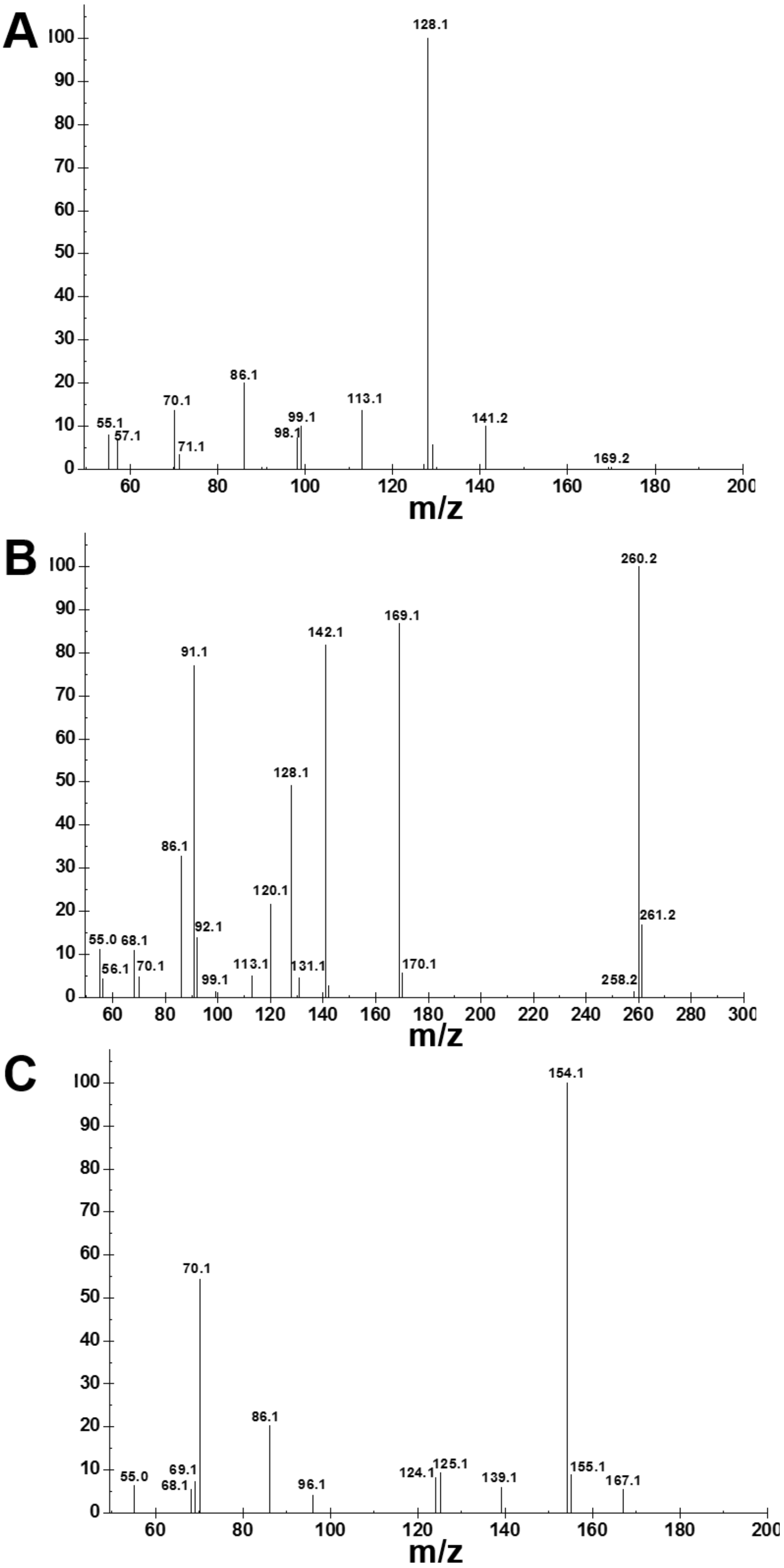

SUPPLEMENTARY FIGURE S2| Structural analysis of three filtrate fractions from starter kimchi inoculated with *Ln. mesenteroides* LBP-K06. (A), (B), and (C) corresponding to KF1, KF2, and KF4, obtained by electron impact using GC-MS. The EI and CI values of

each fraction were suggested in **Table 5**. Together with the mass fragmentation pattern under electron impact, these compounds were assigned to be (A) $C_8H_{12}N_2O_3$, (B) $C_{14}H_{16}N_2O_3$, and (C) $C_{11}H_{18}N_2O_2$, corresponding to cyclo(Ser-Pro), cyclo(Tyr-Pro), and cyclo(Leu-Pro), respectively.

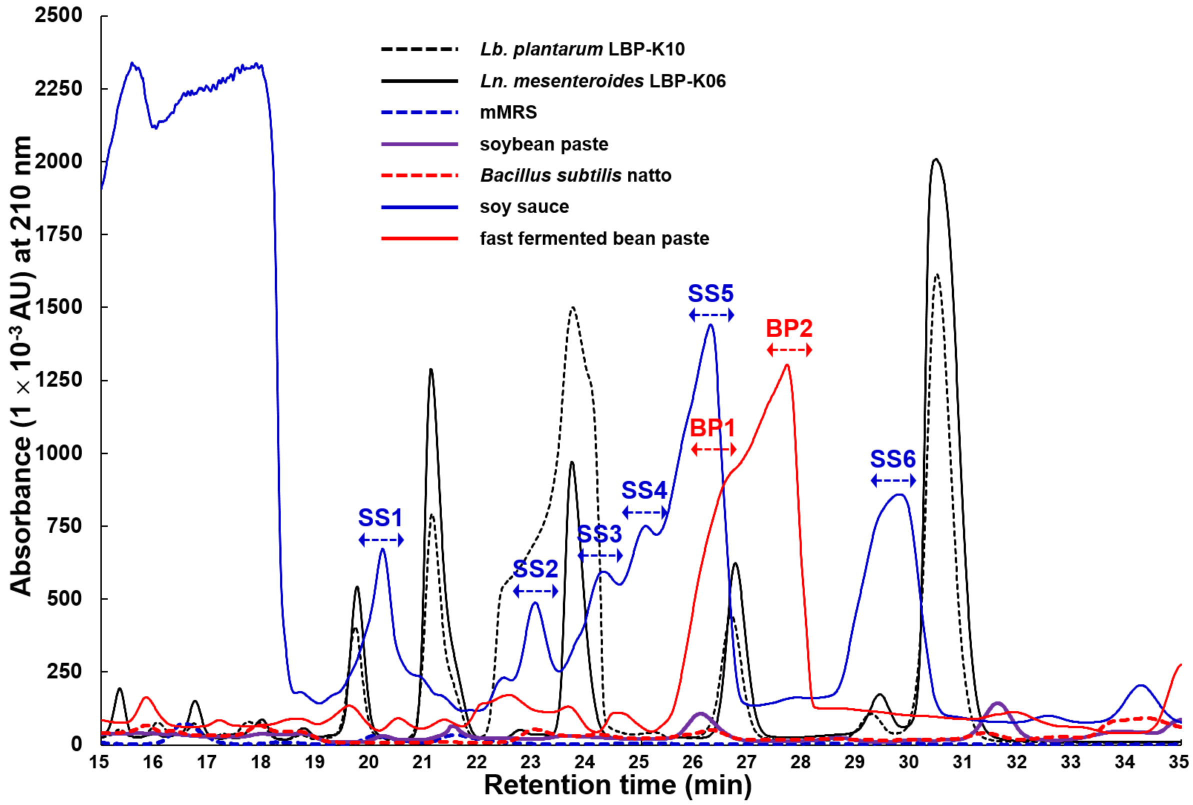

SUPPLEMENTARY FIGURE S3| HPLC fractionation pattern of filtrates from fermented foods of plant origin. The chromatographic separations of whole filtrates from several types of fermented foods of plant origin are shown as described in the materials and methods section. Additionally, culture filtrates in *Lb. plantarum* LBP-K10, *Ln. mesenteroides* LBP-K06, and uninoculated mMRS broth are indicated. A chromatographic analysis of fermented foods of plant origin and culture filtrates derived from LAB strains was performed at a wavelength of 210 nm. Each experiment was performed at least three times independently.

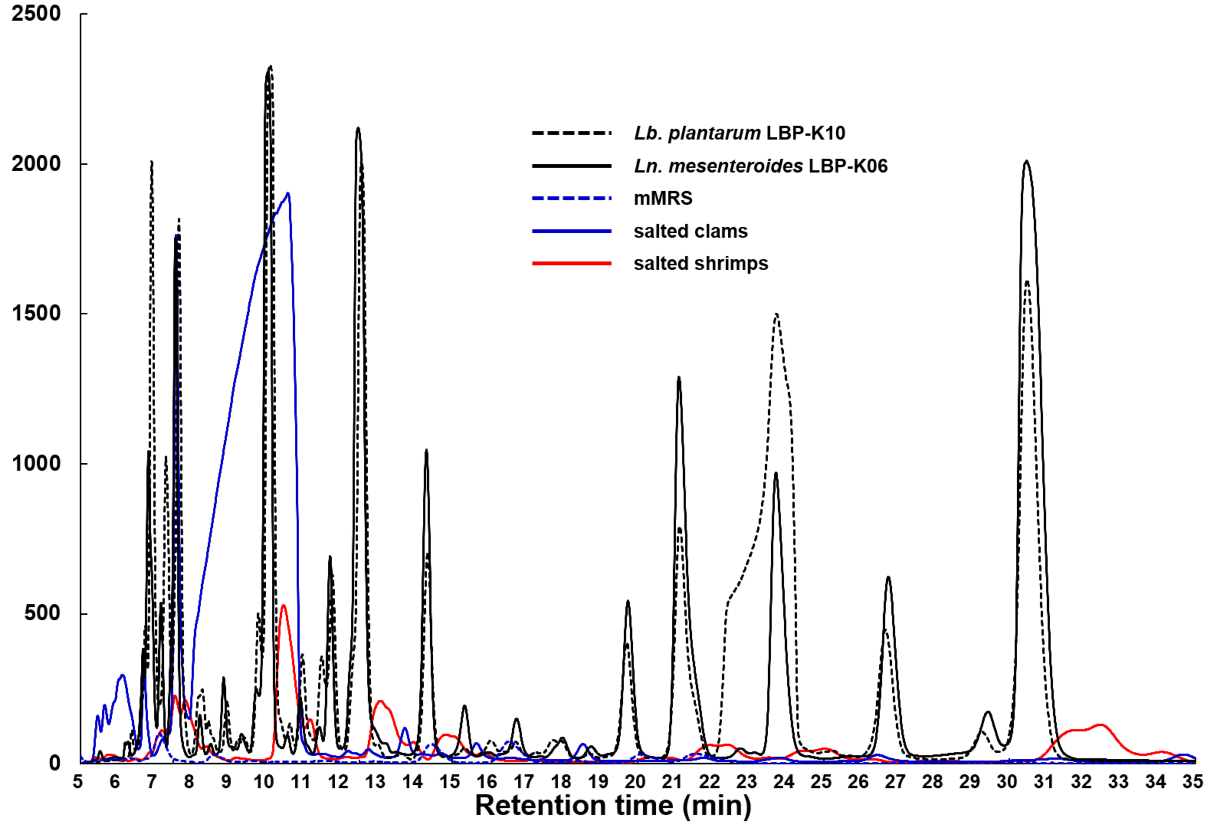

SUPPLEMENTARY FIGURE S4| HPLC analysis of filtrates from fermented foods of animal origin. HPLC fractionation was conducted by using whole filtrates from fermented foods of animal origin. As an earlier experiment, culture filtrates from LAB and uninoculated mMRS broth are used as a reference experiment. A chromatographic analysis of the culture filtrates derived from LAB strains was performed at a wavelength of 210 nm. All experimental trials were repeated at least three times.

#1-1492R_G08:
CCTGATCACCTTCGACGGCTAGCTCCATAAATGGTTACTCCACCGGCTTCGGGTGTTACAAACTCTCG
TGGTGTGACGGGCGGTGTGTACAAGACCCGGGAACGTATTCACCGTAGCATGCTGATCTACGATTAC
TAGCGATTCCAGCTTCATGTAGTCGAGTTGCAGACTACAATCCGAACTGAGAACAACTTTATGGGATT
TGCATGACCTCGCGGTTTAGCTGCCCTTTGTATTGTCCATTGTAGCACGTGTGTAGCCCAAATCATAA
GGGGCATGATGATTTGACGTCATCCCCACCTTCCTCCGGTTTGTCACCGGCAGTCAACCTAGAGTGC
CCAACTAAATGCTGGCAACTAAGTTTAAGGGTTGCGCTCGTTGCGGGACTTAACCCAACATCTCACG
ACACGAGCTGACGACAACCATGCACCACCTGTCACTTTGTCCCCCGAAGGGGAAGGCTCTATCTCTA
GAGTTTTCAAAGGATGTCAAGATTTGGTAAGGTTCTTCGCGTTGCTTCGAATTAAACCACATGCTCCA
CCGCTTGTGCGGGTCCCCGTCAATTCCTTTGAGTTTCAACCTTGCGGTCGTACTCCCCAGGCGGAGT
GCTTAATGCGTTAGCTGCAGCACTAAGGGGCGGAAACCCCCTAACACTTAGCACTCATCGTTTACGG
CGTGGACTACCAGGGTATCTAATCCTGTTTGATCCCCACGCTTTCGCACATCAGCGTCAGTTACAGAC
CAGAAAGTCGCCTTCGCCACTGGTGTTCCTCCATATCTCTGCGCATTTCACCGCTACACATGGAATTC
CACTTTCCTCTTCTGTACT

Blast results (http://blast.ncbi.nlm.nih.gov/)

Staphylococcus equorum **strain +Y18 16S ribosomal RNA gene, partial sequence**

GenBank: JX077101.1

SUPPLEMENTARY FIGURE 5| 16S rRNA sequencing analysis of salted shrimp. The taxonomic identities of the isolates were confirmed by a 16S rRNA gene sequence analysis, and were 99.9% identical to the *S. equorum* type strain, DSM 20674.

Supplementary Tables

SUPPLEMENTARY TABLE S1| LAB strains isolated from traditional Korean fermented plant materials.

	Source and strain number		
	Mustard leaves and stems	Stonecrop	Chinese cabbage
Leuconostoc spp.	93	10	28
Lactobacillus spp.	14	8	17
Lactococcus spp.	—	1	—
Weissella spp.	2	14	18

SUPPLEMENTARY TABLE S2| Antibacterial activities of the culture filtrate isolated from LAB.

Source	Strain	Antagonism test[a]*	MIC[b,c]*	Taxon confirmed by sequencing
Mustard leaves and stems	LBP-B01	++	+++	*Lb. sakei*
	LBP-B02	++	++	*L. kimchii*
	LBP-B03	++	++	*Ln. mesenteroides*
	LBP-B04	++	++	*Ln. mesenteroides*
	LBP-B05	+++	++	*Ln. paramesenteroides*
	LBP-B06	+++	++	*W. cibaria*
Stonecrop	LBP-S01	++	+++	*Lb. sakei*
	LBP-S02	+++	+++	*Lb. plantarum/pentose*
	LBP-S03	++	++	*Lc. lactis*
	LBP-S04	++	++	*L. citreum*
	LBP-S05	++	++	*L. citreum*
	LBP-S06	+	++	*L. lactis*
	LBP-S08	++	++	*W. hellenica*
Chinese cabbage	LBP-K01	++	+++	*Lb. plantarum*
	LBP-K03	++	++	*L. citreum*
	LBP-K04	++	–	*L. citreum*
	LBP-K05	++	++	*L. holzapfelii*
	LBP-K06	+++	++	*Ln. mesenteroides*
	LBP-K07	++	++	*Ln. pseudomesenteroides*
	LBP-K08	++	+	*Ln. mesenteroides*
	LBP-K09	++	++	*Lb. brevis*
	LBP-K10	+++	+++	*Lb. plantarum*
	LBP-K11	++	++	*Ln. citreum*
	LBP-K12	+++	++	*Ln. mesenteroides*
	LBP-K13	++	++	*Ln. mesenteroides*
	LBP-K14	++	++	*Ln. pseudomesenteroides*
	LBP-K15	+++	++	*W. cibaria*
	LBP-K16	++	++	*W. confusa*

[a] Symbol: +, < 15 mm; ++, < 22 mm; +++, > 22 mm (Indicator strain: *B. subtilis)*
[b] MIC: Minimum inhibitory concentration
[c] Symbol: +, 1-fold; ++, 0.5-fold; +++, less than 0.25-fold (Indicator strain: *B. subtilis*)
* All experiments represent the average of three independent experiments.

SUPPLEMENTARY TABLE S3| Relative antibacterial activity of primary HPLC fractions from starter kimchi against bacterial reference strains.

Indicator strains	Relative activity (%)*					
	KF1	KF2	KF3	KF4	KF5	KF6
Gram-positive bacteria						
B. subtilis	11.5	32.6	19.2	91.4	8.4	—
Gram-negative bacteria						
E. coli	9.7	33.9	15.6	100	5.7	—

* The values represent the average of three independent experiments.
